# Librarians collaborating to teach evidence-based practice: exploring partnerships with professional organizations

**DOI:** 10.5195/jmla.2018.341

**Published:** 2018-07-01

**Authors:** Kerry Dhakal

**Affiliations:** Assistant Professor and Research and Education Librarian, Health Sciences Library, The Ohio State University, 376 West 10th Avenue, Columbus, OH 43210

## Abstract

**Objective:**

The study sought to determine if librarians are collaborating with nurses and professional nursing organizations to teach evidence-based practice (EBP) continuing education courses, workshop, classes, or other training activities.

**Methods:**

A 15-question survey was sent to 1,845 members of the Medical Library Association through email.

**Results:**

The survey was completed by 201 consenting respondents. Some respondents (37) reported having experience teaching continuing education in collaboration with professional health care organizations and 8 respondents, more specifically, reported having experience teaching EBP continuing education courses, workshops, classes, or other training activities in collaboration with professional nursing organizations.

**Conclusions:**

The survey results demonstrate that librarians are teaching continuing education classes or workshops in collaboration with professional health care organizations and reveal that there are a small number of librarians collaborating with professional nursing organizations to teach EBP continuing education courses, workshops, classes, or other training activities.

## INTRODUCTION

The Institute of Medicine’s “The Future of Nursing: Leading Change, Advancing Health” report recommends that organizations with a charge for nursing education provide lifelong learning opportunities to nurses [1]. Nurses participate in lifelong learning activities in a range of venues. Professional nursing organizations offer many types of continuing education opportunities to their members, including in-person or online courses, conference workshops, and conference presentations. These opportunities have included continuing education focusing on evidence-based practice (EBP). EBP has been defined as:

a process of using evidence found in clinical trials and research studies, knowledge and information gained from one’s own professional expertise as a practicing clinician, and the consideration of a patient’s concerns and preferences in relation to her/his care to make the best possible decision for the care provided to that patient. [2]

To identify the best available clinical evidence, nurses and other clinicians need to be competent in conducting literature searches and critically appraising evidence from research studies. Many nurses are uncomfortable with searching for research evidence in the literature [3, 4], even though doing so has been identified as one of several competencies for nurses [5, 6]. Based on their assessment of nurses’ perceived value of and ability to implement EBP, Hain and Haras concluded that professional nursing organizations could have a role in promoting EBP [7].

Health sciences librarians regularly teach EBP courses and workshops, assist in developing EBP continuing education course curricula at their home institutions, and help nurses and other clinicians search for evidence in the literature [8–14]. Marshall et al. found the role of libraries in clinical practice and EBP to be valuable to nurses. In their study, 6,788 nurses, among other clinicians, were surveyed about the value of library services. Nurse respondents rated the information found by librarians to be high in quality and cognitive value and to contribute to improved patient care [15]. Allen et al. proposed that nurses and librarians collaborate to exchange knowledge and noted that collaborative programming could be offered at national nursing conferences and marketed to both librarians and nurses [16]. Initiatives by the Nursing and Allied Health Resources Section (NAHRS) and the Hospital Libraries Section (HLS) of the Medical Library Association (MLA) have focused on health sciences librarians’ roles in supporting nurses and nurse researchers who are involved in Magnet status activation and re-designation in hospitals [17, 18].

The primary purpose of this study was to determine the extent to which librarians have partnered with professional health care organizations, particularly professional nursing organizations, as instructors for continuing education courses and other training opportunities. The secondary purpose was to identify librarians’ roles in teaching EBP courses offered by professional nursing organizations.

## METHODS

The institutional review board for behavioral and social sciences at the Ohio State University declared this study exempt from review. The survey instrument was developed to measure if and how librarians have participated as instructors in teaching EBP in collaboration with professional health care organizations, particularly professional nursing organizations.

The survey questions were developed partly based on the author’s own experience teaching EBP to nurses as well as a review of the literature about EBP competencies for nurses [5, 6] and the experience of health sciences librarians in teaching EBP to nurses and other health care professionals [8, 10, 13, 19, 20]. A face validity review of the survey instrument was conducted with the assistance of another health sciences librarian who also teaches EBP prior to the survey being sent to the MLA member list.

MLA is one of the largest professional associations for health information professionals, including librarians; therefore, its membership email list was chosen for distributing the survey. MLA also includes NAHRS, HLS, the Health Association and Corporate Libraries Section, and the Cancer Librarians Section. Thus, the author hoped that the survey would reach many of these section members as well as other MLA member librarians who have taught EBP in collaboration with health care providers or professional organizations. The use of the MLA list was not meant to be comprehensive but was used as a convenience sample that could then be built upon in future studies. Permission was granted by MLA to utilize their member list.

The electronic survey was distributed in April 2017, and a reminder email was sent in May 2017. The survey included fifteen questions (supplemental appendix) and was open for four weeks. The survey design included both closed-ended and open-ended questions [21].

Survey data were collected and analyzed using Qualtrics, the Statistical Package for the Social Sciences (SPSS), and Microsoft Excel. Because most survey questions were multiple choice, the analysis consisted of frequency counts. Other textual data were collected when questions allowed respondents to select “other” as one of the multiple-choice answers and provide narrative information [21].

## RESULTS

The list of MLA members who were asked to participate in the survey included 1,845 individual members, not counting registered guests of the website or organizational members. Eight emails containing a link to the survey instrument were returned as undeliverable. One member answered that they were not a librarian. A total of 202 librarians responded to the survey, providing an 11% response rate.

Of the 202 respondents, 201 consented to the survey. When asked about their membership in professional organizations, 180 respondents answered that they were MLA members. Fifty-nine of those 180 respondents also listed being members of other organizations. Seven additional respondents indicated membership only in professional organizations that were not MLA; 3 of these 7 stated affirmatively that they were members of another professional organization but did not provide the names of those organizations. Fourteen of the 201 consenting respondents did not answer this question.

The professional organizations other than MLA that were listed by survey respondents included international, national, regional, state, and city or local organizations as well as MLA chapters and sections in a variety of practice areas (Table 1). The most frequently listed non-MLA national professional organizations were the American Library Association, the Association of College and Research Libraries, and the Canadian Health Libraries Association/Association des bibliothèques de la santé du Canada.

**Table 1:**
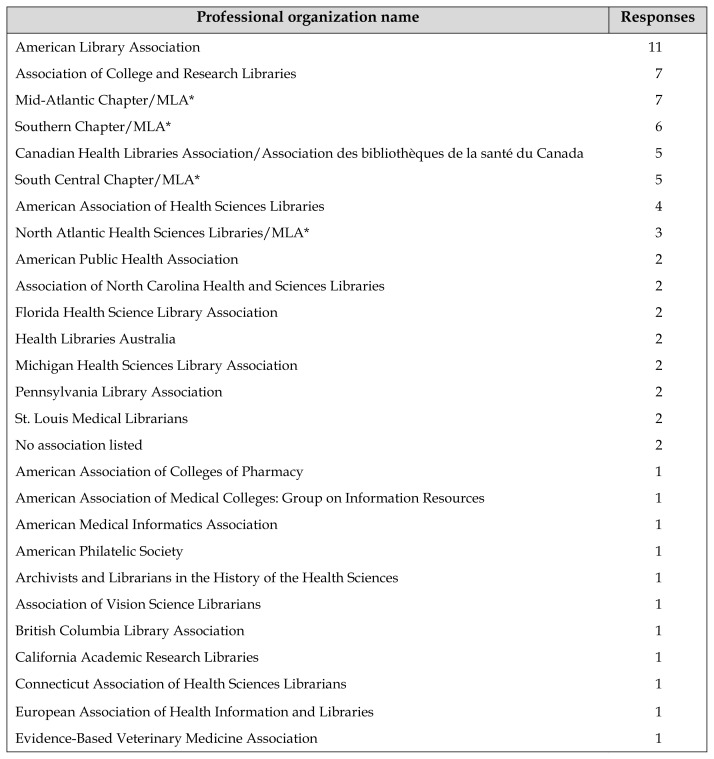
Membership in professional organizations

Professional organization name	Responses
American Library Association	11
Association of College and Research Libraries	7
Mid-Atlantic Chapter/MLA*	7
Southern Chapter/MLA*	6
Canadian Health Libraries Association/Association des bibliothèques de la santé du Canada	5
South Central Chapter/MLA*	5
American Association of Health Sciences Libraries	4
North Atlantic Health Sciences Libraries/MLA*	3
American Public Health Association	2
Association of North Carolina Health and Sciences Libraries	2
Florida Health Science Library Association	2
Health Libraries Australia	2
Michigan Health Sciences Library Association	2
Pennsylvania Library Association	2
St. Louis Medical Librarians	2
No association listed	2
American Association of Colleges of Pharmacy	1
American Association of Medical Colleges: Group on Information Resources	1
American Medical Informatics Association	1
American Philatelic Society	1
Archivists and Librarians in the History of the Health Sciences	1
Association of Vision Science Librarians	1
British Columbia Library Association	1
California Academic Research Libraries	1
Connecticut Association of Health Sciences Librarians	1
European Association of Health Information and Libraries	1
Evidence-Based Veterinary Medicine Association	1
HARL†	1
Health Libraries Inc.	1
Health Science Librarians of Illinois	1
Health Sciences Library Association of New Jersey	1
Kentucky Medical Library Association	1
Massachusetts Health Sciences Libraries Network	1
Medical Library Group of Southern California and Arizona/MLA*	1
Metropolitan Detroit Medical Library Group	1
MHL†	1
Nursing and Allied Health Resources Section/MLA*	1
Ohio Health Sciences Library Association	1
Ontario Health Library Association	1
Pacific Northwest Library Association	1
Special Library Association	1
Tabamlin	1
Texas Library Association	1
Upstate New York and Ontario Chapter/MLA*	1
“Other groups”	1

* MLA chapters were included because sometimes librarians are members of only the chapter.

† The author could not locate the names for these acronyms.

Out of the 201 consenting respondents, 191 answered whether they had any experience teaching or co-teaching a continuing education course, workshop, class, or other training activity for a professional health care organization. Of these 191 respondents, 37 answered that they had this experience, whereas 154 stated that they did not have this experience. Of the 37 respondents who had this experience, 33 provided the names of the professional health care organizations that offered those courses or workshops (Table 2). Five of the 33 respondents listed more than 1 organization. Twelve of the professional health care organizations were national organizations, 6 were state-level organizations, 1 was a city-level organization, and 2 organizations were international organizations. Eleven other respondents listed hospitals, universities, and institutions instead of professional health care organizations; 2 of these 11 respondents listed MLA.

**Table 2:** Experience teaching for a professional health care organization

Professional organization name	Responses
American Public Health Association	5
American Nurses Association	2
American Nurses Credentialing Center	2
Accreditation Council for Continuing Medical Education	1
American Association of Medical Colleges	1
American Congress of Rehabilitation Medicine	1
American Medical Association	1
American Psychological Association	1
Association of Nurses for a Healthy Environment	1
Chicago Public School Nurses	1
Council on Medical Student Education in Pediatrics	1
Evidence-Based Veterinary Medicine Association	1
Idaho Nurses Association	1
Indiana Center for Evidence-Based Nursing Practice	1
Illinois Rural Health Association	1
International Veterinary Emergency and Critical Care Medicine	1
Kentucky Nurses Association	1
National Collaborating Centre for Methods and Tools	1
Organization of Nurse Executives	1
State veterinary medical association	1
Tennessee Nurses Association	1
Other*	11

* Respondents who provided answers that did not match the example of a professional health care organization provided in the survey question.

The 37 librarians who responded that they had experience teaching or co-teaching continuing education coursework for professional health care organizations were prompted to proceed with the survey, whereas the 154 librarians who responded that they did not have this experience were led to the end of the survey and not asked any further questions. Thirty-five of the 37 librarians who responded that they had experience teaching continuing education coursework for professional health care organizations chose to continue with the survey.

When asked if they had taught or co-taught any EBP continuing education courses, workshops, classes, or other training activities for professional nursing organizations, 8 out of 35 respondents indicated having this experience. The remaining 27 respondents answered that they did not have this experience and were led to the end of the survey and not asked any further questions. Seven of the 8 respondents who answered that they had this type of experience provided information about the professional nursing organizations that they collaborated with, including the American Nursing Credentialing Center (n=2), American Nursing Association (n=1), and Kentucky Nurses Association (n=1). Another respondent listed MLA, and 2 others listed educational institutions instead of professional nursing organizations.

The 8 librarians who had experience teaching EBP continuing education coursework for professional nursing organizations were asked about the topics that they taught in these educational sessions. Topics taught included literature searching; patient, intervention, comparison, outcome (PICO) question development; critical appraisal of journals articles; and reference management (Table 3). The survey allowed respondents to choose more than one topic and to list other topics, but none did so.

**Table 3:** Evidence-based practice (EBP) topics taught by librarians

Topics taught	Responses
Literature searching	8
Patient, intervention, comparison, outcome (PICO) question development	6
Critical appraisal of journal articles	5
Reference management	4
Other	0

Librarians were asked if they taught the EBP continuing education courses or workshops alone or with other instructors. The survey allowed the respondents to choose more than one type of instructor and to list other types of instructors, but none did so. Most of the eight librarians indicated that they taught with other instructors, mostly with other librarians, doctoral degree (PhD)–prepared nurses, and nurses with a master of science in nursing (MSN) or master of nursing (MN) degree (Table 4). Only one librarian answered that they taught alone.

**Table 4:** Partnerships with other professionals to teach EBP

Co-instructors of EBP courses	Responses
Another librarian	6
PhD-prepared nurse	4
Nurse with a master’s degree (MSN, MN)	3
Other health sciences professionals	2
Nurse with a bachelor’s degree	1
Registered nurse with no other degrees	1
No one, I was the only instructor	1
Other	0

Respondents were asked about where they taught the EBP continuing education courses or workshops for professional nursing organizations. Respondents could choose multiple answers. Six out of eight respondents answered that they had experience teaching online synchronous courses that were not part of conference offerings, five had experience teaching courses offered in person at an annual conference, three had experience teaching online asynchronous courses, and three had experience teaching in other venues, including an in-person session outside of a conference setting and an in-person six-week course. One respondent listed teaching an elective course but did not state exactly where the course took place.

Of the eight respondents who answered that they had experience teaching EBP continuing education coursework for professional nursing organizations, five answered that they taught one to two courses in total, and three answered that they taught more than six courses, workshops, classes, or other training activities.

Librarians were then asked how they came to teach EBP continuing education coursework for a professional nursing organization. Four of the eight librarians responded that they were contacted by a professional nursing organization to teach an EBP continuing education workshop. Three librarians responded that they took the initiative to learn more about teaching opportunities with professional nursing organizations themselves. One librarian was asked to co-teach a course when a professional nursing organization reached out to a nurse at her institution.

Finally, the survey requested some additional demographic information from the eight librarians who had experience teaching EBP continuing education coursework for professional nursing organizations, including the number of years that respondents had been librarians and their degrees. Four respondents had eleven years of experience or more as librarians, one had seven-to-ten years of experience, three had four-to-seven years of experience, and none had less than three years of experience. All respondents but one had earned a master of library science degree. Additional degrees earned by the librarians included a bachelor of science in nursing, master of science in education, doctorate of education, veterinary technician certification, and PhD in another unidentified field.

## DISCUSSION

Most health sciences librarians teach EBP courses in the context of their role as information experts in academic medical centers or hospitals [8–14]. The audience for such courses may include health sciences students and professional medical and nursing staff members. However, these same health care professionals also seek professional development in EBP education through other venues, such as courses and workshops offered by professional health care organizations, including professional nursing organizations.

The goal of this study was to learn if and how health sciences librarians have partnered with professional health care organizations to teach continuing education courses or workshops and, more specifically, if librarians have taught EBP continuing education courses or workshops in partnership with professional nursing organizations. The results showed that 18% of the 201 consenting respondents partnered with professional health care organizations to teach continuing education. However, only 4% of the same sample of respondents partnered with professional nursing organizations to teach EBP.

Health sciences librarians teaching EBP often teach certain aspects of the process, including developing PICO questions, searching the literature, critically appraising the evidence in journal articles, and developing reference management strategies [8, 10, 11, 19]. They often partner with other nurse educators or nursing faculty to teach these courses, and those partnerships are established by librarians reaching out to nursing faculty or nurse educators and vice versa [8, 12, 13]. The survey results demonstrate that health sciences librarians who teach EBP continuing education courses or workshops for professional nursing organizations also teach these very same topics in partnership with nurse educators, nursing faculty, and other librarians. The results also suggest that both health sciences librarians and professional nursing organizations are taking the initiative to reach out and partner with one another to teach EBP continuing education courses or workshops. In the present study, three health sciences librarians took the initiative to reach out to professional nursing organizations about teaching opportunities, and four professional nursing organizations reached out to health sciences librarians to ask them to teach courses or workshops.

Lastly, health sciences librarians often teach outside of the library setting and in other spaces in their institutions. Eldredge commented on how health sciences librarians have detached themselves from the physical space of the library to accommodate clinical patrons where they work because of the trend of more resources being accessed in digital-only formats [22]. Some health sciences librarians have taken a step further and have become embedded librarians, working solely with a specific discipline or group of clinicians or in a specific unit [19, 23, 24]. According to the results of this study, a few health sciences librarians are taking an even further step of partnering with professional health care organizations and professional nursing organizations.

Limitations of this study include the fact that only health sciences librarians who were listed as MLA members were surveyed. The conclusions, therefore, are not representative of all health sciences librarians. Also, a few respondents listed organizations that were not professional health care or nursing organizations in the narrative sections of the survey, even though examples of organizations were provided in the survey. In any future studies focusing on professional organizations, a clearer definition of organization types should be included in the survey.

Increased collaboration with professional health care organizations and professional nursing organizations could provide yet another platform for health sciences librarians to step out of the library setting and participate in the national agenda to promote and teach EBP competencies.

## SUPPLEMENTAL FILE

Appendix Evidence-based practice continuing education: librarian survey 2017
