# Supplementary material for: Librarians collaborating to teach evidence-based practice: exploring partnerships with professional organizations
Source: J Med Libr Assoc. 2018 Jul 1;106(3):311–9. doi: 10.5195/jmla.2018.341 (PMC6013130; doi:10.5195/jmla.2018.341)
Supplement: Appendix [file jmla-106-311-s001.pdf]

JMLA Journal of the Medical Library Association

SC

Supplemental content to
J Med Libr Assoc. Jul;106(3):dx.doi.org/10.5195/jmla.2018.341
www.jmla.mlanet.org
© Dhakal 2018

Librarians collaborating to teach evidence-based practice: exploring partnerships with professional organizations

Kerry Dhakal, MAA, MLS

APPENDIX

Evidence-based practice continuing education: librarian survey 2017

Default question block

Q1. The Ohio State University Consent to Participate in Research

Study Title: Librarians as Instructors for Professional Nursing Association Evidence-Based Practice Continuing Education Courses, Workshops, Classes, and Other Training Activities

Read the following information and select "I agree" on the bottom of this page if you would like to participate.

This is a consent form for research participation.
It contains important information about this study and what to expect if you decide to participate.

Your participation is voluntary.
You are invited to participate in a research study about evidence-based practice continuing education and training offered by professional health care and nursing organizations and librarians' roles in teaching those courses. Your participation in this research study is voluntary, and you may choose not to participate, or to stop participating, at any time. You may skip any question for any reason. You will not be penalized for not participating or stopping your participation. Risks to you are no more than minimal.

Feel free to ask questions before making your decision about whether or not to participate. If you decide to participate in the survey, you will provide your consent by checking a box at the bottom of this page acknowledging that you have read this information and by submitting your survey responses. No signature or hard copy of this consent is necessary.

What the study is about:
I am collecting information to learn if librarians are collaborating with professional organizations to teach evidence-based practice.

What I will ask you to do:
If you agree to participate in this study, you will be asked to complete a survey that asks about your experience teaching continuing education courses in collaboration with professional health care or nursing organizations (e.g., American Nursing Association, Association of American Medical Colleges).

Once you have consented to participate in a survey, the survey should take 5 minutes to complete. You may skip any question you feel uncomfortable answering. If you decide to stop participating in the project before you submit your results, there will be no penalty to you. The study will maintain anonymity of your participation. Your data will be submitted anonymously and cannot be traced back to you.

Risks and benefits:
No risks are anticipated from participating in this study other than those encountered in day-to-day life. There are benefits to you in that the information gathered in the survey will advance public knowledge

JMLA Journal of the Medical Library Association

SC

Supplemental content to
J Med Libr Assoc. Jul;106(3):dx.doi.org/10.5195/jmla.2018.341
www.jmla.mlanet.org
© Dhakal 2018

and the ongoing conversation in the nursing and health sciences librarianship fields about evidence-based practice education. There is no compensation for this survey.

Confidentiality:
Your answers will be confidential. The records of this study will be kept private. In any report we make public, we will not include information that will make it possible to identify you.

All responses will be used for scholarly purposes. We will work to make sure that no one sees your survey responses without approval. However, because we are using the Internet, there is a small chance that someone could access your online responses without permission. In some cases, this information could be used to identify you.

Participant rights:
You may refuse to participate in this study without penalty or loss of benefits to which you are otherwise entitled. If you are an employee at Ohio State University, your decision and responses will not affect your employment status.

For questions about your rights as a participant in this study or to discuss other study-related concerns or complaints with someone who is not part of the research team, you may contact the Office of Responsible Research Practice.

If you have questions:
The researcher conducting this study is: Kerry Dhakal, and she may be contacted with any questions.

By selecting "I agree" below, I acknowledge that I have read this consent form and am aware that I am being asked to participate in a research study by completing a survey that will begin on the next page. I have had the opportunity to ask questions about this research study and have had them answered to my satisfaction.

O Yes (1)
O No (2)

[Skip To: End of Survey If Q1=No (2)]

Q2. Are you a member of any of these organizations? (Select all that apply.)

O Medical Library Association (MLA) (1)
O Other (2) ____________________

Q3. Have you ever taught or co-taught a continuing education (CE) course, workshop, class, or other training activity offered by a professional health care organization or professional nursing organization (e.g., American Medical Association, American Nursing Association)?

If yes, please provide a list of the organization(s).

O Yes (1) ____________________
O No (2)

[Skip To: End of Survey If Q3=No (2)]

JMLA Journal of the Medical Library Association

SC
Supplemental content to
J Med Libr Assoc. Jul;106(3):dx.doi.org/10.5195/jmla.2018.341
www.jmla.mlanet.org
© Dhakal 2018

Q4. Have you ever taught or co-taught a continuing education course, class, or workshop for a professional nursing association about evidence-based practice?

If yes, please provide a list of the organization(s).
O Yes (1) ____
O No (2)

[Skip To: End of Survey If Q4=No (2)]

Q5. What aspects of evidence-based practice did you teach? (Select all that apply.)
O Patient, intervention, comparison, outcome (PICO) question development (1)
O Literature searching (2)
O Reference management (3)
O Critical appraisal of journal articles (4)
O Other (5) ____

Q6. Who else taught the course(s) with you? (Select all that apply.)
O PhD-prepared nurse (1)
O Doctor of nursing practice (DNP)–prepared nurse (2)
O Nurse with a master's degree (3)
O Nurse with a bachelor's degree (4)
O Registered nurse with no other degrees (5)
O Another librarian (6)
O Other health sciences professionals (7)
O Other (8) ____
O No one, I was the only instructor (9)

Q7. At what venues have you taught evidence-based practice continuing education (CE) courses, classes, or workshops for professional nursing organizations? (Select all that apply.)
O In person CE courses at annual conferences (1)
O Virtual CE courses at annual conferences (2)
O Online, synchronous courses, classes, or workshops (outside of a conference setting) (3)
O Online, asynchronous courses, classes, or workshops (outside of a conference setting) (4)
O Other venue (5) ____

Q8. In what year(s) below did you teach evidence-based practice course(s), class(es), or workshop(s) for a professional nursing organization?
O 2017 (1)
O 2016 (2)
O 2015 (3)
O 2014 (4)
O 2013 (5)
O 2012 (6)
O Before 2012 (7)

JMLA Journal of the Medical Library Association

SC

Supplemental content to
J Med Libr Assoc. Jul;106(3):dx.doi.org/10.5195/jmla.2018.341
www.jmla.mlanet.org
© Dhakal 2018

Q9. How many evidence-based practice CE courses, classes, or workshops have you taught for professional nursing organizations in total?
O 1–2 (1)
O 3–4 (2)
O 5–6 (3)
O More than 6 (4)

Q10. Have you ever presented a podium presentation about evidence-based practice at a professional nursing organization's annual conference?
O Yes (1)
O No (2)

Q11. How did you become an instructor or co-instructor for a professional nursing organization?
O I contacted a professional nursing organization about teaching opportunities (1)
O A professional nursing organization reached out to me to ask for my participation (2)
O I presented a podium presentation with a person or team from my institution and learned about this opportunity (3)
O I work for a professional nursing organization (4)
O Other (5) ____________________

Q12. Where do you work?
O In the United States (1)
O Outside of the United States (2)

Q13. How long have you been a librarian?
O 0–3 years (1)
O 4–7 years (2)
O 7–10 years (3)
O 11 years or more (4)

Q14. What degrees have you earned? (Select all that apply.)
O MLS or MLIS (1)
O RN with no other degree (2)
O BSN (3)
O MSN or MN (4)
O DNP (5)
O PhD in nursing (6)
O PhD in another field (7)
O Other (8) ____________________

Q15. What type of library do you work in?
O Academic health sciences library (1)
O Hospital library (2)
O Professional nursing association (3)
O Professional health care association (non-nursing) (4)
O General academic library (5)
O Other type of library (6) ____________________
